# Quality assurance as an integral component of diagnostic testing in clinical laboratories and point-of-care testing: The Uganda experience

**DOI:** 10.4102/ajlm.v5i2.447

**Published:** 2016-10-17

**Authors:** Steven Aisu, Wilson Nyegenye, Victor Bigira, Charles Kiyaga, Michael Dfendu, Sam Acellam, Richard Walwema, Lydia Nakiyingi, Isaac Sewanyana, Aida Namakula, Richard Batamwita, William Lali

**Affiliations:** 1 Central Public Health Laboratory, Ministry of Health, Kampala, Uganda; 2 Clinton Health Access Initiative, Kampala, Uganda; 3 Infectious Disease Institute, Kampala, Uganda; 4 World Health Organization, Kampala, Uganda

## HIV situation in Uganda

**FIGURE 1:**
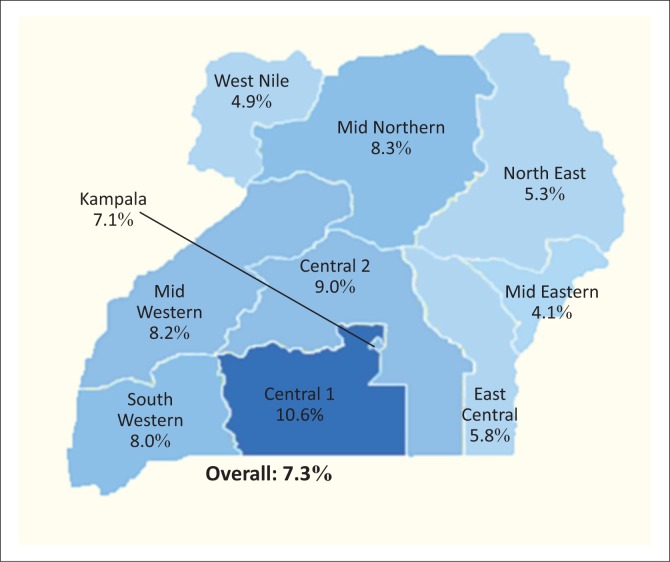
Key HIV statistics in Uganda, 2014, showing HIV prevalence by region.

Uganda, with an estimated population of 35 million people, is a landlocked country that forms part of the East African Union member states. The country is divided into nine political regions (Figure 1), in which there are 112 administrative districts. In order to make the administration and coordination of health services more efficient, the country is further divided into 14 health regions.

Uganda is classified as a high burden HIV country with an estimated 1.6 million people living with HIV/AIDS and 140 000 new cases reported annually (Table 1; Figure 1).^1^ Being one of only two countries where incidence was on the rise in 2013, HIV remains a major public health problem in Uganda.^2^ HIV prevalence varies across the country, being highest in the Central region and near urban centres and lowest in the mid-eastern and West Nile regions.

**TABLE 1:** Key HIV statistics in Uganda, 2014.

Key statistic	Value
Country population	~35 million
New HIV infections	140 000 per annum
People living with HIV/AIDS	1.4 million
HIV-positive pregnant women	100 000 per annum
HIV-positive babies born	16 000 per annum
Conventional CD4 machines	200
Point-of-care CD4 machines	300
Access to CD4 testing	60% – 65%
Centralised early infant diagnosis testing	
- % of PMTCT sites	81%
- -[Reach]	~53%
- Turnaround time (sample to results)	~11 days

PMTCT, prevention of mother-to-child transmission.

In 2013, the World Health Organization changed the recommendation for initiation of antiretroviral therapy from a CD4 count of 350 cells/mm^3^ to 500 cells/mm^3^.^3^ Uganda was one of the first countries to adopt these recommendations and began implementing the revised treatment guidelines in April 2014.^4^ Under the revised antiretroviral therapy guidelines, CD4 enumeration remains critical to staging the need for antiretroviral therapy among HIV-positive patients aged 15 years and above.^5^ In addition, the World Health Organization recommended the use of viral load testing as the preferred antiretroviral therapy monitoring approach. The Ministry of Health in Uganda subsequently adopted viral load monitoring for antiretroviral therapy patients into the national guidelines. Uganda’s viral load programme has since scaled up rapidly, but universal access has not yet been achieved. For patients who do not have access to viral load services, CD4 testing remains essential for monitoring antiretroviral therapy response. As Uganda scales up the new World Health Organization/UNAIDS 90-90-90 strategy,^6^ there has been a steep rise in the number of people in need of HIV testing and viral load monitoring across the country. It is estimated that over 850 000 people will be on antiretroviral therapy by the end of 2016. Meeting these targets will require innovative ways of developing laboratory systems, including scaling up of point-of-care (POC) tests for HIV testing, diagnosis of opportunistic infections and monitoring response to treatment or disease progression.

## Uganda’s laboratory infrastructure and HIV-related testing services

The laboratory network in Uganda consists of a five-tiered system covering the following levels: national, regional, general hospital (district), health centre IV and health centre III. Laboratories at national, regional and district levels are relatively well developed, whereas laboratories at lower health centre IV and III level generally have inadequate space, no running water, frequent power interruptions and inadequate working space. In order to overcome some of these challenges, the country has established a national sample and results referral network based on hubs. A hub is a high volume laboratory that functions as a referral laboratory for 30–40 lower level laboratories. Complex tests that cannot be handled at the hub, such as early infant diagnosis (EID), viral load testing, culture for tuberculosis and outbreak investigation samples, among others, are centralised at the Central Public Health Laboratory (CPHL) and other reference laboratories.^7,8,9^

Although testing capacity has been increased through deployment of modern high-throughput equipment, national testing targets for EID and viral load have not yet been met. It can be argued that lack of, or limited access to, laboratory services may be overcome through centralisation of testing services and building of new laboratories. However, this course of action requires substantial investment and could result in a significant delay. Moreover, there is a nagging fear that if donor funding is significantly reduced or entirely stopped, such projects may be rendered ‘white elephants’. Cognisant of such possibilities, over the last few years, Uganda has adopted a combination of conventional testing platforms at higher level health facilities and POC testing at lower level health facilities. Thus, in Uganda, EID and viral load testing use Roche Molecular Diagnostics and Abbott Molecular laboratory methods, while CD4 and HIV testing use a mix of conventional analysers and POC devices, including BD FACSCalibur^®^, BD FACSCount^®^, Partec CyFlow^®^ Counter and Alere Pima™ for CD4 counts, and ELISA and rapid diagnostics tests for HIV. In addition, BD MGIT is used for tuberculosis cultures and Cepheid GeneXpert^®^ for PCR. POC tests are deployed in remote rural areas or where test volumes are low, while conventional analysers are used mainly at hubs and in urban areas.

## Quality assurance in Uganda

### Current quality assurance situation

The quality assurance programme in Uganda is still in its infancy. Like many African countries, Uganda has adopted the Strengthening Laboratory Management Towards Accreditation and Stepwise Laboratory Quality Improvement Process Towards Accreditation approaches to improving quality systems in laboratories. However, the supporting components to this programme, such as internal quality control procedures and external quality assessment (EQA), are quite weak. Although CPHL has put in place some measures in collaboration with the Uganda Virus Research Institute, the National Tuberculosis and Leprosy Control Program, the Infectious Diseases Institute and the National Drug Authority to carry out some EQA functions, major gaps still exist. There is poor EQA coverage, particularly for POC testing; low response rates from participating facilities and lack of a comprehensive corrective action plans to support poorly performing laboratories. Only panels for HIV testing and tuberculosis smear microscopy are produced locally, whereas panels for clinical chemistry, haematology, bacteriology, CD4 counts and viral load are supplied by external agencies. The EQA schemes and suppliers are not centrally coordinated. Because Uganda anticipates a rapid scale up of POC testing, CPHL has developed plans to review the POC quality assurance policies and framework.

## Laboratory and point-of-care quality assurance policy and framework in Uganda

### Current status of point-of-care quality assurance policy and framework

Uganda has a National Health Laboratory Policy and Strategic Plan that guides the implementation of Laboratory Quality Management systems across the health laboratory services. However, the strategies do not explicitly address POC testing deployment or POC testing quality issues. Therefore, there is a need to review the National Laboratory Policy and Strategic Plan so as to address quality systems for POC testing.

Table 2 shows the results of a rapid assessment of the POC testing quality assurance policy and framework. While there is top-level leadership engagement (estimated score 80%), major gaps still exist for coordination at the national level (60%), definition of roles and responsibilities (60%), policies and strategic plans (40%), and availability of National Laboratory Standards (30%).

**TABLE 2:** Status of point-of-care quality assurance policy and framework in Uganda

Area	Status	Remarks	Est. Score
Top level Leadership engagement	Clear buy-in by all management and leadership levels about the role of POC testing in healthcare	There is a need to provide updates about POC testing; need for sensitisation on regional and international standards and ministerial declarations on POC testing and to seek additional resources.	80%
Coordination at national level	Quality assurance office staffed by a national quality assurance officer at CPHL and supported by quality assurance TWG as a sub-committee of LTC	The quality assurance office requires additional staff; Accreditation (1), Basic LQMS (1), EQA (1).	60%
Definition of roles and responsibilities	Regional quality committees are in place. Their role is coordination of quality efforts in the districts (7–10) within their catchment areas; there are quality focal points in facilities	The regional quality assurance networks require further strengthening. There is still doubt in many labs as to where responsibility for quality falls.	60%
Policy and Strategic plan	A National Health Laboratory Policy, NHL Strategic Plan, NHL M&E plan and Quality master plan exist	There is no specific policy statement on POC testing. There is a need to update the policies and plans to provide specific reference to POC testing.	40%
National Laboratory Standards	Minimum standards for laboratories have been set.	The country has not yet set minimum standards that focus on POC testing. There is a need to review manufacturers’ performance and specifications versus performance in the field for each POC test and then set goals and standards.	30%

CPHL, Central Public Health Laboratory; EQA, external quality assurance; LTC, National Health Laboratories Technical Committee; LQMS, Laboratory Quality Management System; M&E, monitoring and evaluation; NHL, National Health Laboratory; POC, point-of-care; TWG, technical working group.

### External quality assurance reforms for point-of-care testing

**FIGURE 2:**
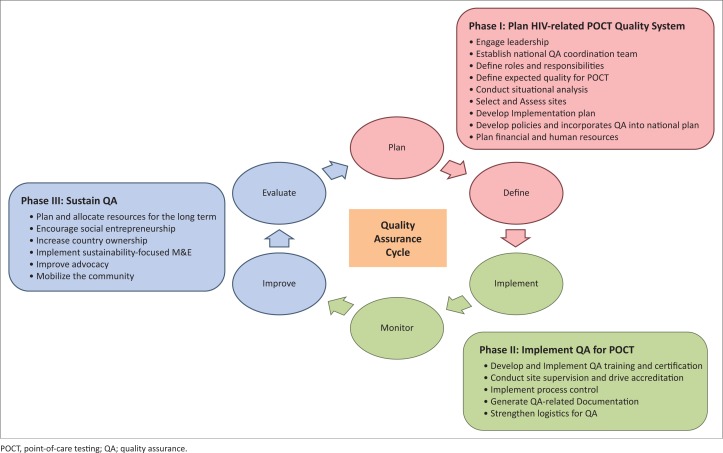
Components of a functional EQA scheme.

A functional EQA scheme consists of six key components (Figure 2), specifically: plan, define, implement, monitor, improve and evaluate. These components are arranged in a cyclical manner to demonstrate the concept of continual improvement.

In Uganda, implementation of the EQA scheme will be done in three phases, as outlined below.

#### Phase 1: Plan an HIV-related point-of-care testing quality system

**FIGURE 3:**
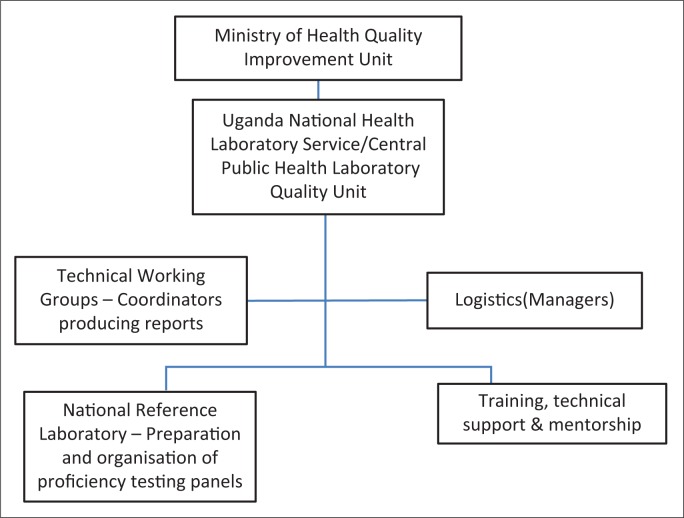
Proposed national external quality assessment coordination structure.

Planning and developing an HIV-related POC quality system requires the review of the current guidelines and standards, and therefore requires the engagement of relevant leaders to execute the activities. With leadership engagement, establishment of the national quality assurance coordination team is easier and the roles and responsibilities of the selected teams can be defined (Figure 3). Before defining the expected quality of POC tests, there is a related need to conduct a situational analysis that may include mapping disease prevalence, as well as selecting and assessing sites. With the abovementioned background activity, the implementation plan will be developed with the further incorporation of quality assurance and policies. Finally, a financial plan will be drafted with an emphasis on human resources and cost–benefit analysis.

#### Phase 2: Implement quality assurance for point-of-care testing

**FIGURE 4:**
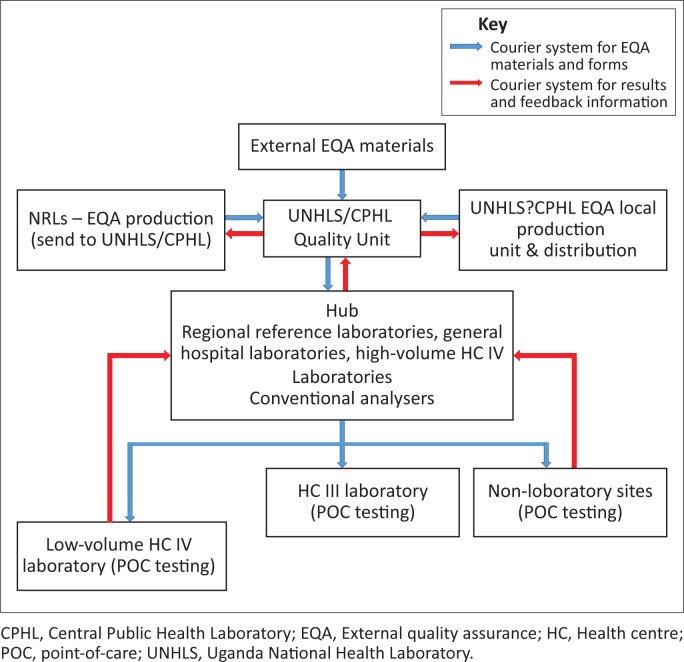
Proposed courier system for the flow of national external quality assessment materials, forms, results and feedback.

The implementation strategy for quality assurance of POC testing requires quality assurance training and certification. This will further involve activities such as the process control, audits, site support supervision and mentorship, all of which constitute the fundamental components of capacity building. Establishment of EQA (active, intermediate, passive) production, packaging/shipping through hubs, programme management and outsourcing will be implemented when the need arises (Figure 4). The quality assurance supply chain and logistics programme can continuously be strengthened for improvement.

#### Phase 3: Sustain the quality assurance programme

For the sustainability of the quality assurance programme planning, allocation of resources and continuous encouragement of socio-economic entrepreneurship are required. Sustainability implementation will require focused monitoring and evaluation initiatives, country ownership and involvement, a high level of advocacy, and mobilisation of the community. Feedback, corrective action and inter-lab comparisons are vital for quality assurance programmes, as these are the basis for improvement.

#### Cost of activities and resources for quality assurance improvement

Estimating the cost of EQA activities will include understanding the country’s needs based on identified interventions to inform key policy decisions and reducing high costs. External support will be sought to price the final model of service provision and to establish a cost-effective POC testing EQA scheme that is integrated in a comprehensive National EQA Program. In cases where it will be necessary to produce EQA panels locally, the activity will be undertaken by the Uganda National Health Laboratory and respective reference laboratories. EQA materials will be distributed using the hub system. Technical assistance and other resources will be mobilised both internally and externally to support the programme.

### Conclusion and way forward

Uganda is actively working to implement a robust quality assurance programme that will address POC testing in the country. Although the quality assurance programme is still in its infancy, Uganda has already adopted Strengthening Laboratory Management Towards Accreditation and Stepwise Laboratory Quality Improvement Process Towards Accreditation to improve overall laboratory quality systems and is focusing on how to improve internal quality control procedures and EQA.

Uganda has always taken great strides to meet challenges with innovative approaches. The country established a national sample and results referral network based on hubs to improve EID services, which also resulted in cost savings for the programme and in turn improved other health services. More recently, Uganda performed a rapid assessment of the POC testing quality assurance policy and framework to identify major gaps that should be addressed.

Uganda understands the strength of working in a collaborative manner with all institutions to implement a sustainable country-owned EQA programme. CPHL is working with the Uganda Virus Research Institute, the National Tuberculosis and Leprosy Control Program, the Infectious Diseases Institute and the National Drug Authority to plan and carry out EQA functions necessary to support quality CD4, EID, and viral load services.

## References

[1] Joint United Nations Programme on HIV/AIDS (UNAIDS) The gap report. Geneva, Switzerland: UNAIDS; 2014.

[2] Ministry of Health, Macro I Uganda AIDS Indicator Survey (AIS) 2011. Kampala, Uganda; Ministry of Health; 2011.

[3] World Health Organization WHO issues new HIV recommendations calling for earlier treatment [page on the Internet]. c2013 [cited 2016 Sep 22]. Available from: http://www.who.int/mediacentre/news/releases/2013/new_hiv_recommendations_20130630/en/

[4] Ministry of Health, Uganda Addendum to the national antiretroviral treatment guidelines [document on the Internet]. c2013 [cited 2016 Sep 27]. Available from: http://preventcrypto.org/wp-content/uploads/2012/07/Uganda-National-ART-Guidelines_2014.pdf

[5] Ministry of Health, Uganda Uganda national policy guidelines for HIV counselling and testing [document on the Internet]. c2012 [cited 2016 Sep 22]. Available from: http://www.who.int/hiv/pub/guidelines/uganda_art.pdf

[6] Joint United Nations Programme on HIV/AIDS (UNAIDS) 90-90-90: An ambitious treatment target to help end the AIDS epidemic. Geneva, Switzerland: UNAIDS; 2014.

[7] PEPFAR, USAID Increasing viral load monitoring of people living with HIV on ART in Northern Uganda in line with the 90-90-90 global targets [document on the Internet]. c2016 [cited 2016 Sep 27]. Available from: https://www.usaidassist.org/sites/assist/files/improving_vl_testing_in_northern_uganda_june2016_a4_ada.pdf

[8] Kiyaga C, Sendagire H, Joseph E, et al Uganda’s new national laboratory sample transport system: a successful model for improving access to diagnostic services for Early Infant HIV Diagnosis and other programs. PLoS One. 2013;8(11):e78609 http://dx.doi.org/10.1371/journal.pone.0078609. eCollection 2013. 24236026 10.1371/journal.pone.0078609 PMC3827263

[9] Stevens WS, Gelman R, Glencross DK, et al Evaluating new CD4 enumeration technologies for resource-constrained countries. Evaluating diagnostics: the CD4 guide. Nature Rev Microbiol. 2008;6(11 Suppl):S29–S38. http://dx.doi.org/10.1038/nrmicro2000 10.1038/nrmicro2000 22745957

